# Effect of gamma irradiation on the Cu(II), Ru(III) and Pd(II) complexes of azo antipyrine moiety and their biological applications

**DOI:** 10.1038/s41598-025-22252-3

**Published:** 2025-10-28

**Authors:** Ehab M. Abdalla, Samar A. Aly, Ayman E. Hassan, Safaa S. Hassan, Emad F. Newair, Shimaa Hosny

**Affiliations:** 1 Chemistry Department, Faculty of Science, New Valley University, Alkharga, 72511 Egypt; 2 Department of Environmental Biotechnology, Genetic Engineering and Biotechnology Research Institute, University of Sadat City, Sadat City, 32958 Egypt; 3 Chemistry Department, Faculty of Science, Cairo University, Giza, 12613 Egypt; 4 Chemistry Department, Faculty of Science, Sohag University, Sohag, Egypt

**Keywords:** Complex, Irradiation, Antibacterial, Anticancer, Molecular docking, Cancer, Chemistry

## Abstract

Three novel Cu(II), Ru(III), and Pd(II) chelates of the ligand N-(1,5-dimethyl-3-oxo-2-phenyl-2,3-dihydro-1H-pyrazol-4-yl)carbonohydrazonoyl dicyanide (HL) were synthesized and fully characterized using IR, NMR, elemental analysis, molar conductivity, melting point, mass spectra, electronic spectra, PXRD, and thermal analysis. Geometry optimization with Gaussian09 confirmed the structures: [Cu(HL)Cl(OH)].H_2_O (B1), [Ru(HL)Cl_2_(OH)(H_2_O)].H_2_O (B2), and [Pd(HL)Cl_2_].2H_2_O (B3). The antibacterial and anticancer activities of non-irradiated and γ-irradiated complexes were evaluated against multiple bacterial strains and the MCF-7 breast cancer cell line. The results demonstrated that most of the tested compounds possess broad-spectrum bactericidal activity. The Cu(II) complex (B1) exhibited the highest anticancer activity, followed by Ru(III) (B2), Pd(II) (B3), and the free ligand HL. Moreover, after irradiation, both the ligand and its complexes became more active against tumor cells. Molecular docking studies supported these results, revealing strong interactions with target proteins responsible for microbial and cancer inhibition. This study highlights the novelty of the metal–HL complexes and shows how γ-irradiation can influence their biological behavior. After irradiation, the complexes displayed stronger activity against cancer cells but a reduced effect on bacteria and fungi, pointing to their potential use as selective multifunctional therapeutic agents.

**Supplementary Information:**

The online version contains supplementary material available at 10.1038/s41598-025-22252-3.

## Introduction

Azo dye compounds have established a vital position as ligands in coordination chemistry due to their attractive chemical and physical properties and wide range of applications in various scientific domains. Many researchers have focused their attention on its compounds, as it contains a –N = N– group, because they serve as effective complexing agents, forming a variety of complexes with various transition metals^1,2^. Their metal complexes are essential for organic syntheses and catalysis, but they also have a variety of biological, pharmacological, and analytical applications^3^. Ampyrone is an antipyrine derivative with an amino group at position C-4 that serves a variety of biological purposes. It is often referred to as 4-Aminoantipyrine^4,5^, their compounds are crucial to contemporary organic synthesis because they are of significant biological significance in addition to being a particularly practical class of heterocyclic compounds^6^. The chemistry of transition metals with 4-aminoantipyrine complexes has garnered a lot of attention due to their potential applications as functional materials and in bioinorganic chemistry, as well as their rational design and manufacture in coordination chemistry^6,7^**,** and used as an anti-cancer, anti-bacterial, anti-fungal and antibiotic^8^. Moreover, Gamma irradiation is known to induce structural and electronic modifications in coordination compounds, which can significantly influence their physicochemical and biological properties^9^. Irradiation can alter particle size, crystallinity, oxidation states, and surface activity, thereby enhancing catalytic, antimicrobial, and anticancer performance^10^. Previous reports have demonstrated that γ-irradiation can improve the antibacterial activity of metal complexes by increasing membrane interactions and generating reactive oxygen species. In addition, irradiation may lead to subtle structural rearrangements that strengthen metal–ligand coordination or improve drug–protein binding, thus enhancing therapeutic potential^11^. Numerous investigations showed how exposure to γ-irradiation changed the antibacterial activity of many tested materials^12,13^. The literature has disclosed numerous Copper (II), Ruthenium (III), and Palladium (II) complexes exhibiting diverse biological effects, such as antibacterial and anticancer properties. The Cu(II) combination was discovered to have potent antibacterial action against the bacterial species under investigation as well as breast cancer cell lines^14–16^, It was additionally discovered that bacterial activity improves after irradiation. Also for palladium and ruthenium complexes^17^. When ampicillin and gentamicin were administered as usual medications, the Ru(III) complex was more effective against some bacterial species. The complexes shown intriguing anticancer potential when tested against the breast cancer cell line MCF-7^18^. The synthesis, characterization, and bioactivities of a Copper (II), Palladium (II) and Ruthenium (III) chelates with antipyrine derivative ligand are discussed below, along with the impact of gamma irradiation.

## Materials and methods

Comprehensive details regarding the supplies, apparatuses, and methods utilized in relation to structural validation and proposal can be found in the supplemental file (Section S1) antimicrobial treatment^19–21^. (Section S2): anticancer activity^22,23^.

### Synthesis of HL ligand

The ligand was synthesized by coupling the diazaonium salt of 4-aminoantipyrine with malononitrile in sodium hydroxide solution. The product was recrystallizes several times with ethanol^24^.

### Synthesis of chelates

**Fig. 1:**
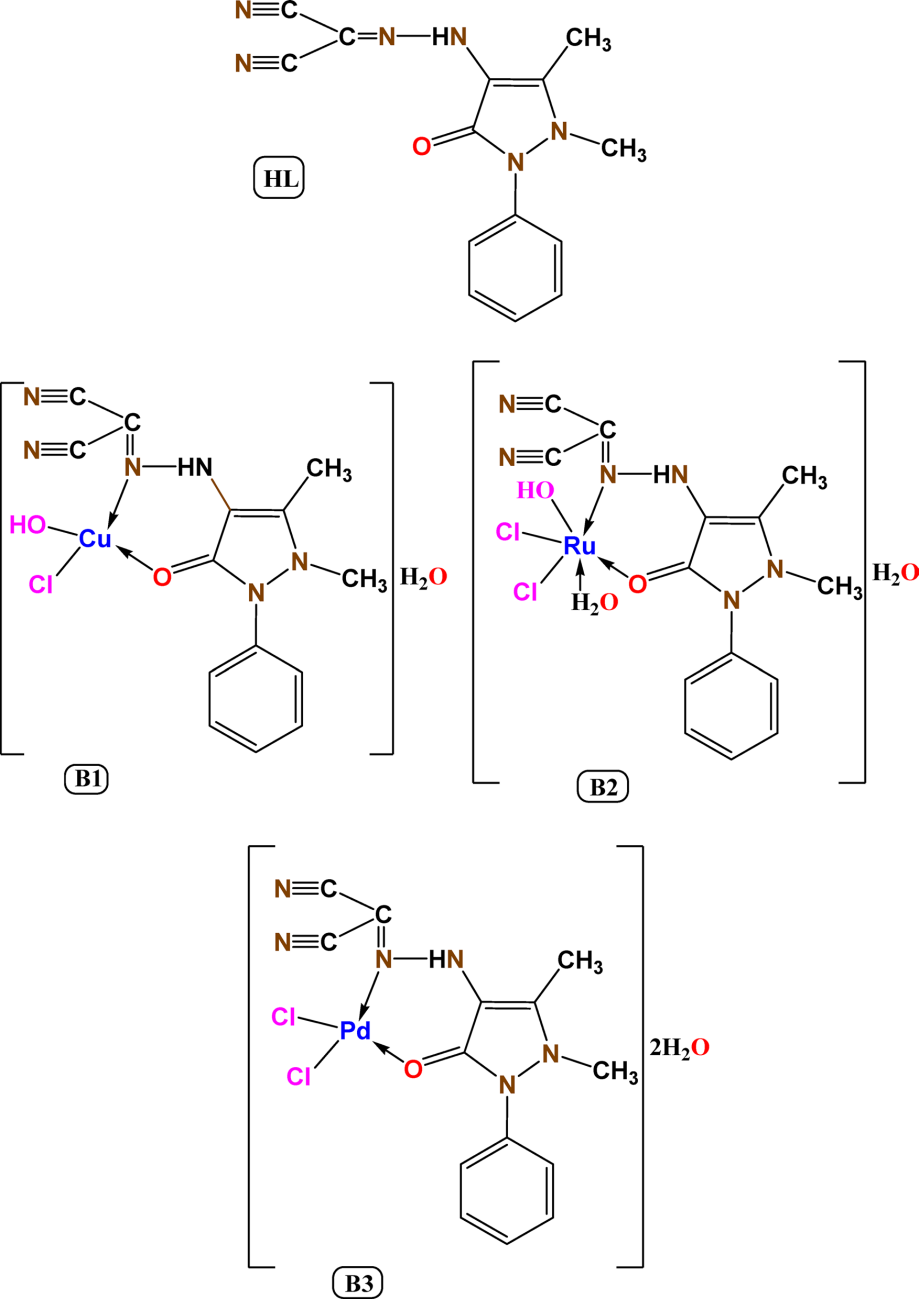
The chemical structure of HL and its metal chelates.

The metal chelates are synthesized by stirring magnetically the ethanolic solutions of 0.002 mol at 60 °C for the solutions of CuCl_2._2H_2_O, RuCl_3._3H_2_O and K_2_PdCl_4_ with the 0.002 mol of the appropriate ligand for periods 4–6 h^25^. The resultant particles were removed by filtering, repeatedly cleaned with EtOH, and vacuum-dried over P_4_O_10_ (Fig. 1**)**.

## Results and discussion

### Physico-chemical characterization

The complexes’s analytical outputs validate the creation of 1:1 (M:L) complex, Table 1**,** and are consistent with the molecular formulas proposed (Fig. 1). The molar conductivity value of B1, B2 and B3 are 19, 26, 21 Ω^−1^cm^2^mol^−1^ in DMF solution that supported their non-electrolytic nature^26,27^.

**Table 1:** Analytical data of the compounds.

No	Compounds	Color Yield %	Molecular weight	Conductivity µs	M. P. °C	Cal. (Found) %			
						C	H	N	M
HL	C_14_H_12_N_6_O	Orange 84	280.11	–	151	59.99 (59.56)	4.32 (4.28)	29.98 (29.91)	–
B1	C_14_H_15_ClCuN_6_O_3_ [Cu(HL)Cl(OH)].H_2_O	Green 79	414.31	19	> 300	40.59 (40.51)	3.65 (3.69)	20.28 (20.16)	15.34 (15.31)
B2	C_14_H_17_Cl_2_N_6_O_4_Ru [Ru(HL)Cl_2_(OH)(H_2_O)].H_2_O	Deep brown 81	505.30	26	> 300	33.28 (33.24)	3.39 (3.41)	16.63 (16.61)	20.00 (19.88)
B3	C_14_H_16_Cl_2_N_6_O_3_Pd [Pd(HL)Cl_2_]0.2H_2_O	Orange 81	493.64	21	> 300	34.06 (33.98)	3.27 (4.01)	17.02 (16.99)	21.56 (21.54)

### ^1^H -NMR spectra

**Fig. 2:**
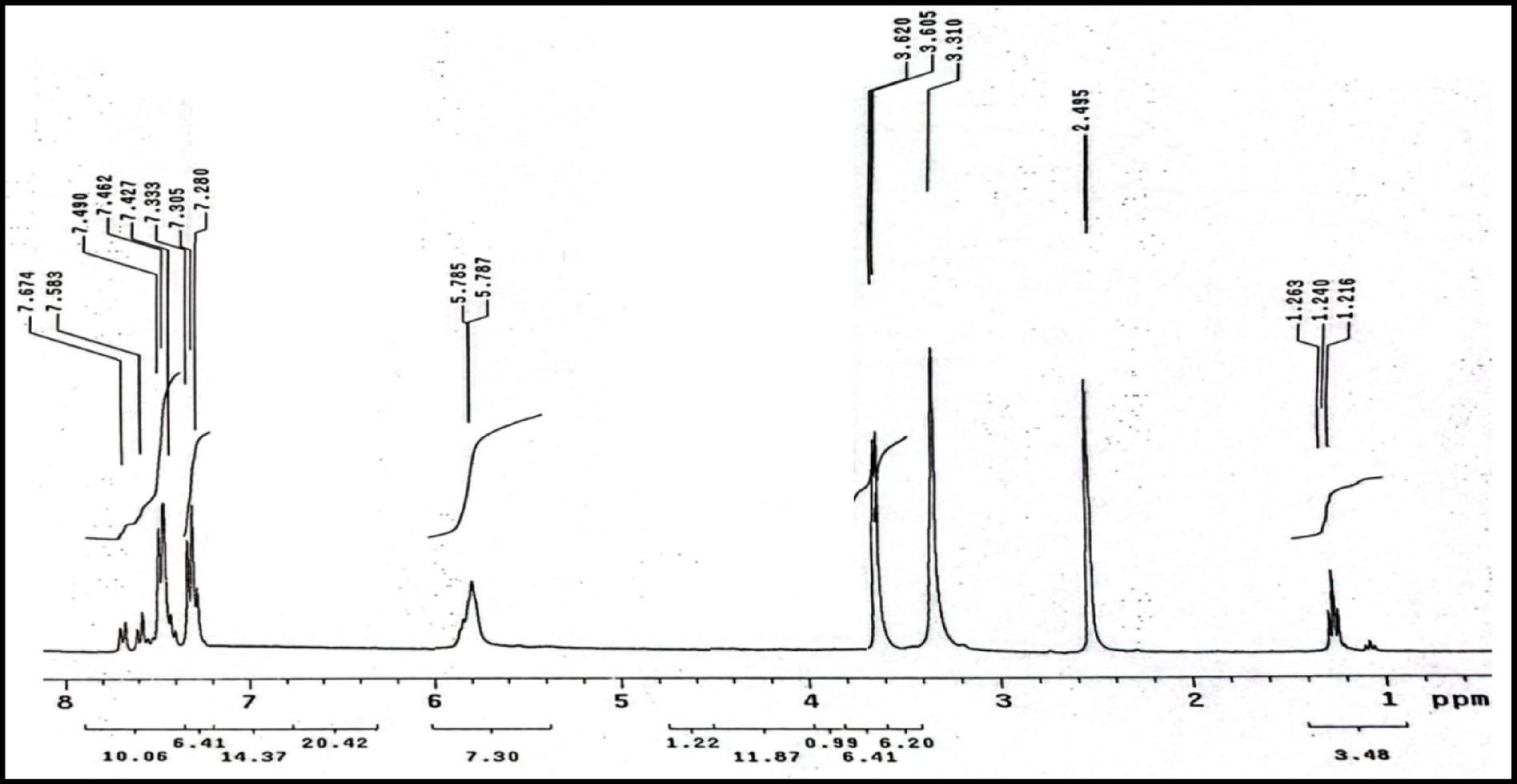
^1^H-NMR spectrum of ligand.

^1^H-NMR spectrum of (HL) in DMSO-d_6_ revealed the characteristic signals to each proton type as observed in (Fig. 2). The observed chemical shifts (δ/ppm) are present at 2.49 (d, 3H, C–CH_3_), 3.31–3.62 (s, 3H, N—CH_3_), 5.78 (s, 1 H, NH), and 7.28–7.67 (aromatic protons)

### FT-IR spectra of compounds

The FTIR values that represent irradiated (A) and non-irradiated (B) Table 2 clarify the most characteristic FTIR values of all compounds in both investigated cases (A) for the irradiated state and (B) for the non-irradiated state. B and A spectra represented bands at (3201); (2206, 2211); (1635, 1633) and (1589) cm^−1^ which corresponded to the υ(N–H), υ (C≡N), υ (C=O), and υ (C=N) for the non-irradiated and irradiated states respectively. Upon complexation, a clear shifts are observed related to (C=O), and υ (C=N) groups as seen in Table 2 that supported the coordination via the azomethine and carbonyl groups. Strong bands at ((3433 &3438), (3442), (3449 &3465)) are ascribed to υ(OH)/H_2_O for Cu(II), Ru(III), Pd(II) chelates, respectively. The new bands exhibited at (548, 565, 589, 594), (452, 459, 467, 468, 471, 479) related to ν (M–O) and ν(M–N), respectively^23,28^. We notice that after gamma radiation, some bands frequencies decreased and others increased. The infrared spectra for irradiated HL and chelates revealed changes in the size and intensity of the bands with the action of irradiation in all compounds^12^.

**Table 2:** Most characteristic values of FT-IR analysis for compounds.

No	Compounds	υ(OH)/H_2_O	υ(N–H)	υ (C=O)	υ (C=N)	υ (M–O)	υ (M–N)
B	HL	–	3201	1635	1589	–	–
A		–	3201	1633	1589	–	–
B1	[Cu(HL)Cl(OH)].H_2_O	3433	3059	1648	1581	548	452
A1		3438	3212	1600	1559	565	459
B2	Ru(HL)Cl_2_(OH)(H_2_O)].H_2_O	3442	3202	1632	1552	589	467
A2		3442	3201	1632	1554	589	468
B3	[Pd(HL)Cl_2_].2H_2_O	3449	3092	1605	1558	594	471
A3		3465	3061	1596	1558	594	479

### Electronic transitions and magnetic properties.

**Fig. 3:**
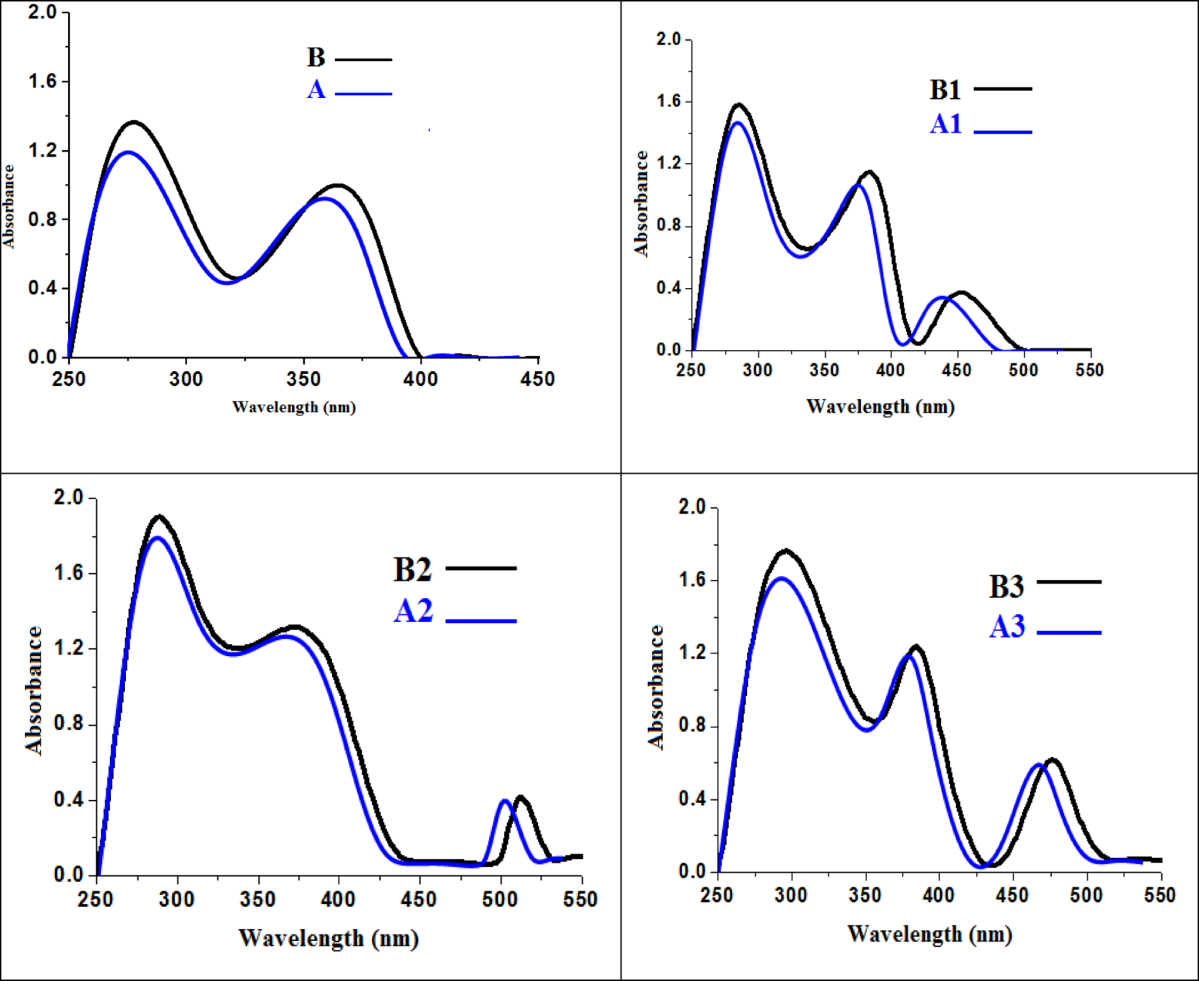
UV–vis transitions of all compounds.

The UV–Vis spectra of the HL and chelates before and after irradiation were obtained in DMSO at room temperature. Table 3 presents the transition values for the maximum absorption wavelength (λ_max_) and the magnetic moments (μ_eff_) while the spectra are shown in Fig. 3. There are two absorption bands visible in the ligand’s (B and A) absorption spectra. The initial great intensity band seen at λ_max_ = (266 and 261) nm may be caused by the aromatic rings’ π → π* transition. The second bands at (378 and 374) nm are caused by charge transfer and the n → π* transition of the azomethine group (C=N). The electronic spectra of the Cu(II), Ru(III), Pd(II) complexes demonstrated that the azomethine nitrogen was coordinated to the metal ions, as evidenced by bands that were displaced to three band before and after irradiation in the range (278—286); (381—393) and (449 —509) nm for the π-π*, n-π* and d-d transitions, respectively, when compared to those of the free ligand, it is notice absorption bands of the wavelength in the ligand strongly changed with the emergence of new d-d transition for all metal complexes^29–31^, where the bands are shifted with about (15–20) nm in wavelength. The electronic spectra of the Cu(II) complexes displayed bands at (449 and 439) nm, which can be assigned to a d–d transition (2Eg → 2T2g) consistent with a distorted octahedral geometry^32^. The Ru(III) complexes exhibited bands at (509 and 501) nm, attributed to ^2^T2g → ^2^A2g transitions of low-spin d^5^ octahedral systems^33^. For the Pd(II) complexes, the absorptions at (482 and 471) nm corresponds to ^1^A1g → ^1^A2g/^1^Eg transitions typical of square-planar d^8^ complexes^34^. These assignments support the proposed geometries obtained from spectral and computational analyses. The magnetic values of Cu(II) and Ru(III) complexes are 1.98 and 1.77 B.M. were assigned to the tetrahedral and octahedral geometry, respectively. While Pd(II) complex showed a diamagnetic character which were assigned to the square planar geometry. Following γ-irradiation, every peak shown in the spectral diagram was noted. The variation in the electronic transitions of all compounds was examined regarding the λ_max_ and absorbance values, revealing no alteration in the geometry of the complexes; this finding is consistent with earlier related research. Irradiation can cause disruption of energy levels along with a distortion in the molecule^9,12^.

**Table 3:** The uv–vis transitions and magnetic moment values of all compounds before and after irradiation.

No	Compound	Wavelength λ_max_ (nm)	Assignment	μ_eff_ (BM)
B	C_14_H_12_N_6_O	266	*π*-*π* ∗	–
		378		
A		261		
		374	n-*π* ∗	
B1	C_14_H_15_ClCuN_6_O_3_	281	*π*-*π* ∗	1.98
		393		
		449		
A1		279	n-*π* ∗	
		381	d-d	
		439		
B2	C_14_H_17_Cl_2_N_6_O_4_Ru	279	*π*-*π* ∗	1.77
		390		
		509		
A2		278	n-*π* ∗	
		388	d-d	
		501		
B3	C_14_H_16_Cl_2_N_6_O_3_Pd	286	*π*-*π* ∗	0.0
		388		
		482		
A3		284	n-*π* ∗	
		385	d-d	
		471

### ESI–MS spectra

**Fig. 4:**
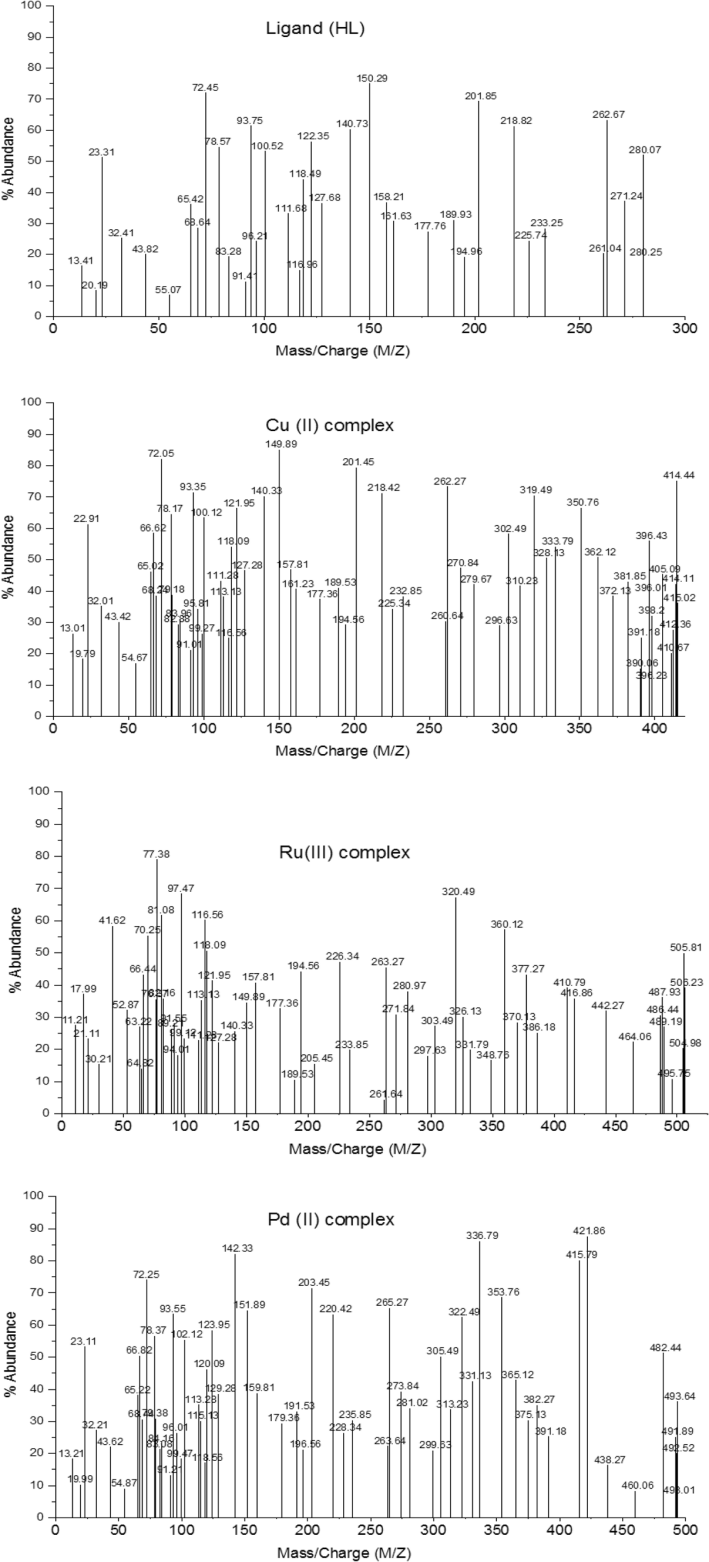
Mass spectra of HL and chelates.

The use of MS to clarify the molecular structure of HL and chelates. Figure 4 observed the ESI–MS spectra of the HL and the Cu(II), Ru(III), Pd(II) complexes. The supposed degradation pattern for complexes and ligand is displayed in Figure S1. Molecular ion peaks are visible in the mass spectra of ligand and Cu(II), Ru(III), Pd(II) complexes at m/z 280.07, 414.44, 505.81 and 493.64 amu for HL and chelates, respectively. The suggested molecular formulae m/z (280.11, 414.31, 505.30 and 493.64) amu, respectively, and this data agree well.

### X-ray diffraction

**Fig. 5:**
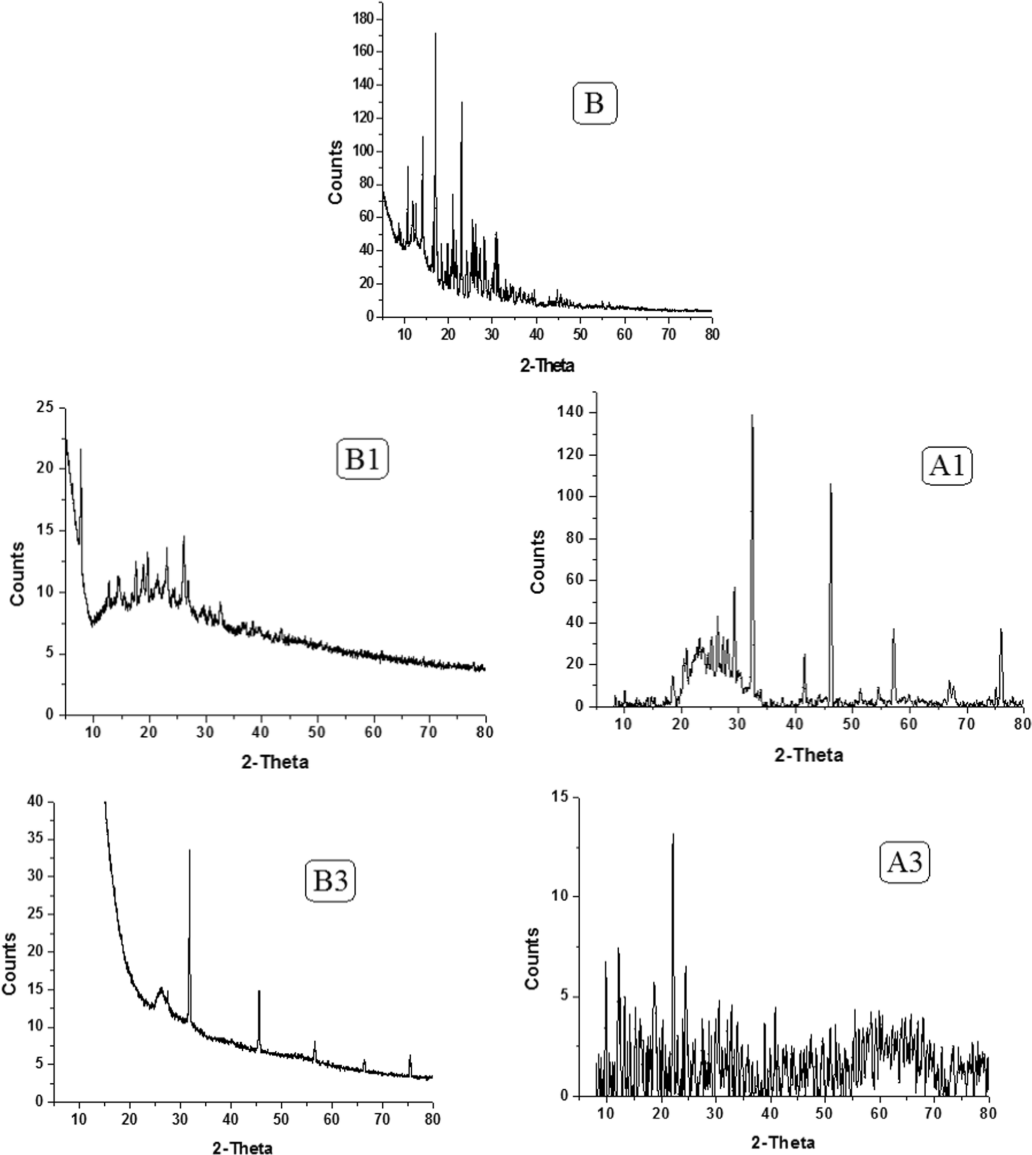
PXRD powder pattern of ligand and complexes before and after irradiation.

**Fig. 6:**
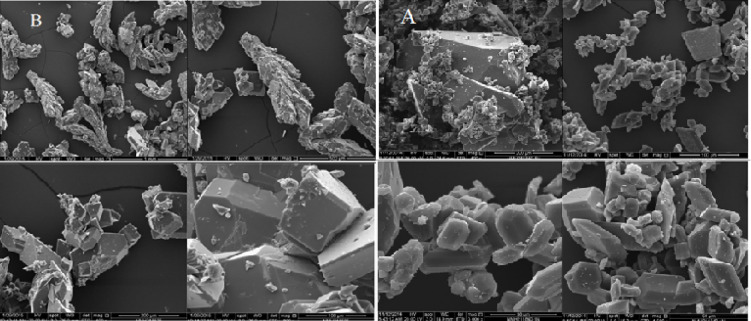
SEM of ligand before (**B**) and after (**A**) irradiation.

**Fig. 7:**
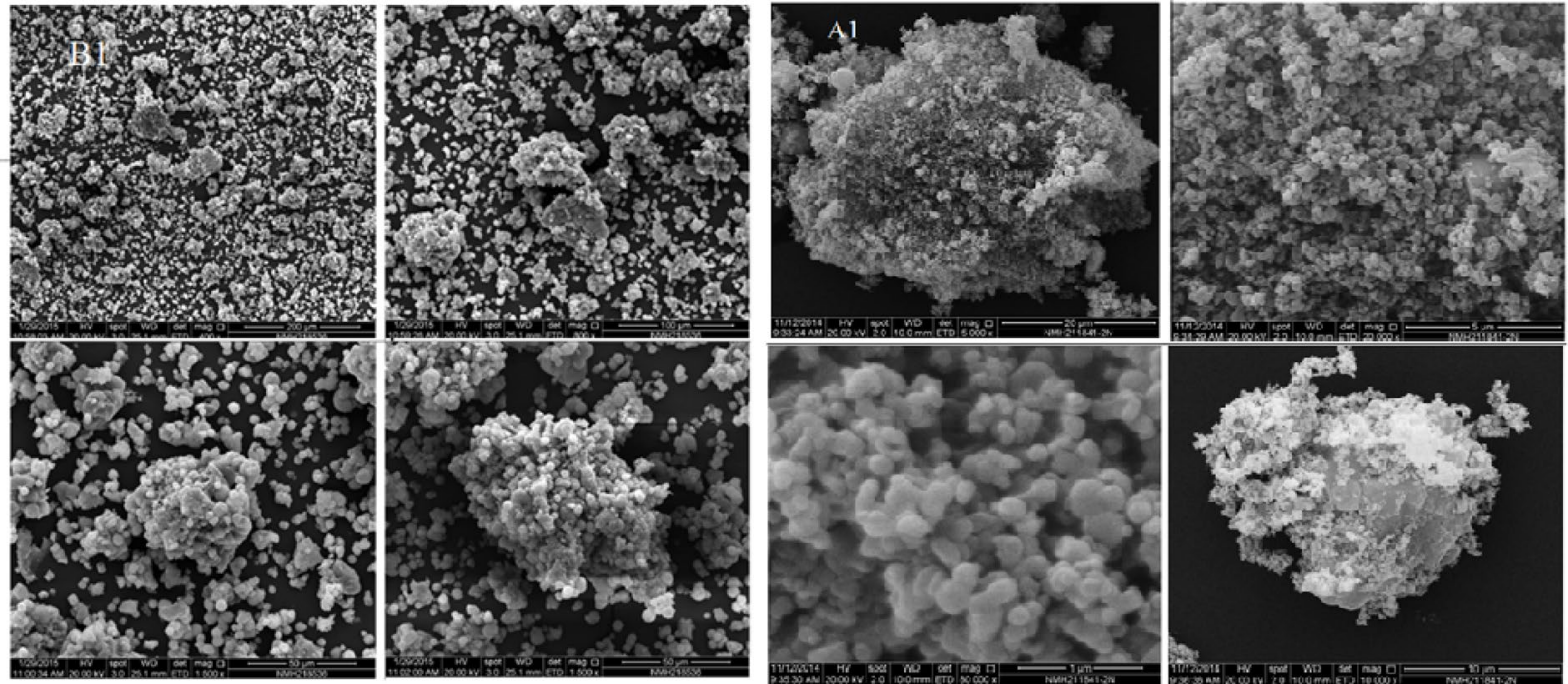
SEM of Cu(II) complex before (**B1**) and after (**A1**) irradiation.

**Fig. 8:**
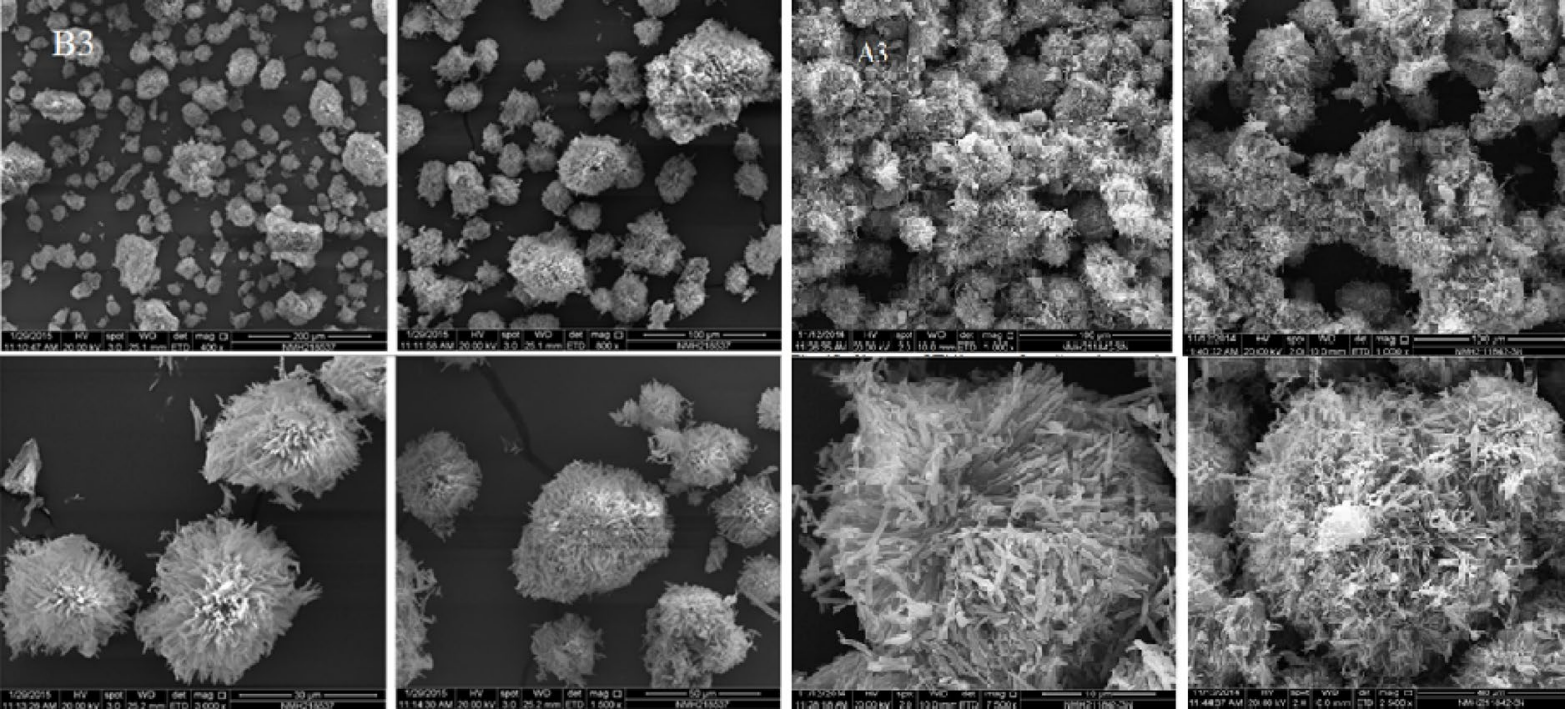
SEM of Pd(II) complex before (**B3**) and after (**A3**) irradiation.

Since the single crystal development was unsuccessful, all compounds underwent powder X-ray diffraction (PXRD) analysis. Figure 5 shows the PXRD of the ligand and complexes combination over 2θ = 5–80 °. Well-defined crystalline peaks were visible in the patterns. Using the Scherer Equation ^35–37^, the average particle sizes of all compounds are represented in Table 4, all compounds observed nano structure size. The crystallinity index values (CI) (Table 4) are calculated employing the equation CI = A_crystal_/A_total_, where A_crystal_ and A_total_ denote the diffraction peak areas of the crystalline area and the overall area, respectively. Scanning electron microscopy (SEM) was utilized to describe the surface structure of ligand, Cu(II) and Pd(II) compounds, as shown in Figs. 6, 7 and 8, in order to make the spherical shape of each chelate more evident. Every chelate was effectively synthesized at the nanoscale.

**Table 4:** PXRD values for compounds**.**

No	Compounds	Crystal size(nm)	Crystallinity index
B	HL	5.45	83.5
B1	[Cu(HL)Cl(OH)].H_2_O	4.03	93.9
A1		14.97	86.59
A2	[Ru(HL)Cl_2_(OH)(H_2_O)].H_2_O	7	20.51
B3	[Pd(HL)Cl_2_]0.2H_2_O	7.92	97.5
A3		29.9	65.42

### Thermogravimetric analysis

**Fig. 9:**
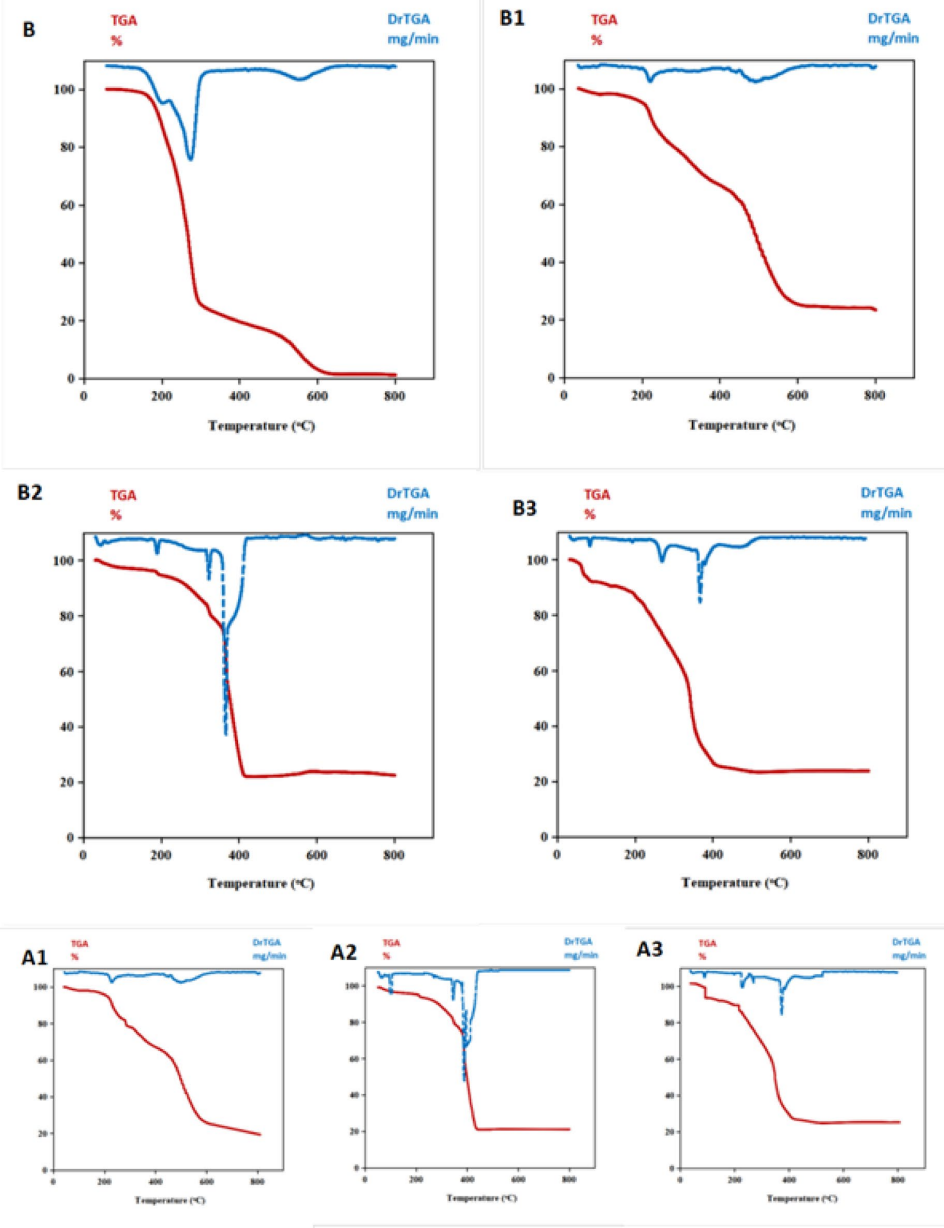
TGA/ DTG curves of the synthesized compounds before and after irradiation.

The behavior of the thermals was investigated using thermogravimetric techniques of the compounds combination during a temperature range of 25 to 800 °C. The outcomes are displayed in Table 5 and Fig. 9. The ligand was shown to be partially decomposed at temperatures between 98 and 425 °C, and fully decomposed at temperatures over 425 °C.

**Table 5:** Thermal values of all non-irradiated and irradiated compounds.

No	Compound	TGA ^o^C	Wt. loss Calc.(Found)%	Leaving species
	C_14_H_12_N_6_O Residue	98–425	83.15 (83.26)	Decomposition of the partial of organic ligand
		> 425	16.84 (16.74)	Complete decomposition of the organic ligand
B1	C_14_H_15_ClCuN_6_O_3_ Residue	20–188.5	4.34 (4.24)	H_2_O
		188.5–430	32.13(32.02)	C_7_H_5_ON_2_
		430–601	41.42(41.38)	C6H_8_N_4_Cl
		> 601	22.10(22.16)	CuO + C
A1		20–212	4.34 (4.14)	H_2_O
		212–462	35.03(34.96)	C8H_5_ON_2_
		462–599	41.42(41.37)	C6H_8_N_4_Cl
		> 599	19.20(19.30)	CuO
B2	C_14_H_17_Cl_2_N_6_O_4_Ru Residue	20–150	10.49 (10.53)	2H_2_O hy.,coord. + OH^−^
		150–320	5.95 (6.13)	C_2_H_6_
		320–495	25.43 (25.49)	C_3_HClN_4_
		396–590	34.95 ( 34.92)	C_9_H_5_ClN_2_
		> 590	23.17 (23.14)	RuO
A2		20–102	10.49 (10.19)	2H_2_O hy.,coord. + OH^−^
		102–220	5.15 (5.21)	CN^−^
				C_9_H_5_ClN_2_
		220–380	34.95 ( 34.45)	C_4_H_7_ClN_3_
		380–410	26.24 (27.01)	RuO
		> 410	23.17 (23.14)	
B3	C_14_H_16_Cl_2_N_6_O_3_Pd Residue	20–145	14.68 (14.25)	2H_2_O hy. + HCl
		145–470	5.88 (5.98)	C_2_H_5_
		470–620	15.62 (15.72)	C_6_H_5_
		620–623	39.02 (40.12)	C_6_HClN_6_
		> 623	24.80 (24.85)	PdO
A3		20–198	7.30 (7.53)	2H_2_O hy
		198–320	10.54 (10.52)	C_2_N_2_
		320–396	12.87 (12.68)	C_2_H_4_Cl
		396–521	44.49 (44.45)	C_10_H_8_ClN_4_
		> 521	24.80 (24.82)	PdO

#### Cu (II) chelates before and after irradiation

The thermograms of the chelates (B1 and A1) were three steps apart, where the first weight at 20–212 °C loss is (Calc./Found %: 4.34/4.24 and 4.34/4.14) which agreed to the loss of hydrated water. The second decomposition of (B_1_ and A_1_) was with weight loss (Calc./Found %: 32.13/32.02 and 35.03/34.96), which corresponded to the dissociation of C_7_H_5_ON_2_ and C8H_5_ON_2_ relative to non-irradiated and irradiated compound, respectively at 188.5–462 °C. The third step indicated the removal of C_6_H_8_N_4_Cl with weight loss (Calc./Found%: 41.42/41.38 and 41.42/41.37). The final residual step observed CuO + C and CuO through the unirradiated and irradiated Cu(II) complexes, respectively^38^.

#### Ru(III) chelates before and after irradiation

The TGA curves of Ru(III) chelates (B2 and A2) showed four steps for losing weight (Calc./Found%: 10.49/10.53 and 10.49/10.19), related to the evaporation of different types of water molecules with (OH) ligand within the heat at 20–150 and 20–102 °C. The 2nd step demonstrated the decomposition due to the heating range 150–320 and 102–220 °C with losing weight (Calc./Found %: 5.95/6.13 and 5.15/5.21) corresponded to C_2_H_6_ and CN. The 3rd step within 320–495 and 220–380 °C for the elimination of C_3_HClN_4_ and C_9_H_5_ClN_2_ with weight loss (Calc./Found%: 25.43/25.49 and 34.95/34.45). The fourth step demonstrated the emergence of the degradation due to the temperature from 396–590 and 380–410 °C with losing weight (Calc./Found %: 34.95/34.92 and 26.24/27.01) corresponded to C_9_H_5_ClN_2_ and C_4_H_7_ClN_3_. leaving RuO as a residue.

#### Pd(II) chelates before and after irradiation

The TGA curves of Pd(II) chelates (B3 and A3) showed four steps for losing weight (Calc./Found%: 14.68/14.25 and 7.30/7.53), allocated to the liberation of two water molecules in a hydrated state + HCl molecules within the heat at 20–145 and 20–198 ^°^C. The second stage revealed the emergence of the breakdown due to the heat from 145–470 and 198–320 ^o^C with losing weight (Calc./Found %: 5.88/5.98 and 10.54/10.52 ) corresponded to C_2_H_5_ and C_2_N_2_. The 3rd step within 470–620 and 320–396 ^°^C indicated the elimination of C_6_H_5_ and C_2_H_4_Cl with weight loss (Calc./Found%: 15.62/15.72 and 12.87/12.68). The fourth step within 620–623 and 396–521 ^°^C with losing weight (Calc./Found %: 39.02/40.12 and 44.49/44.45) corresponded to C_6_HClN_6_ and C_10_H_8_ClN_4_ leaving PdO as a residue^9^.

### DFT calculation

#### Molecular DFT calculation of ligand B

**Fig. 10:**
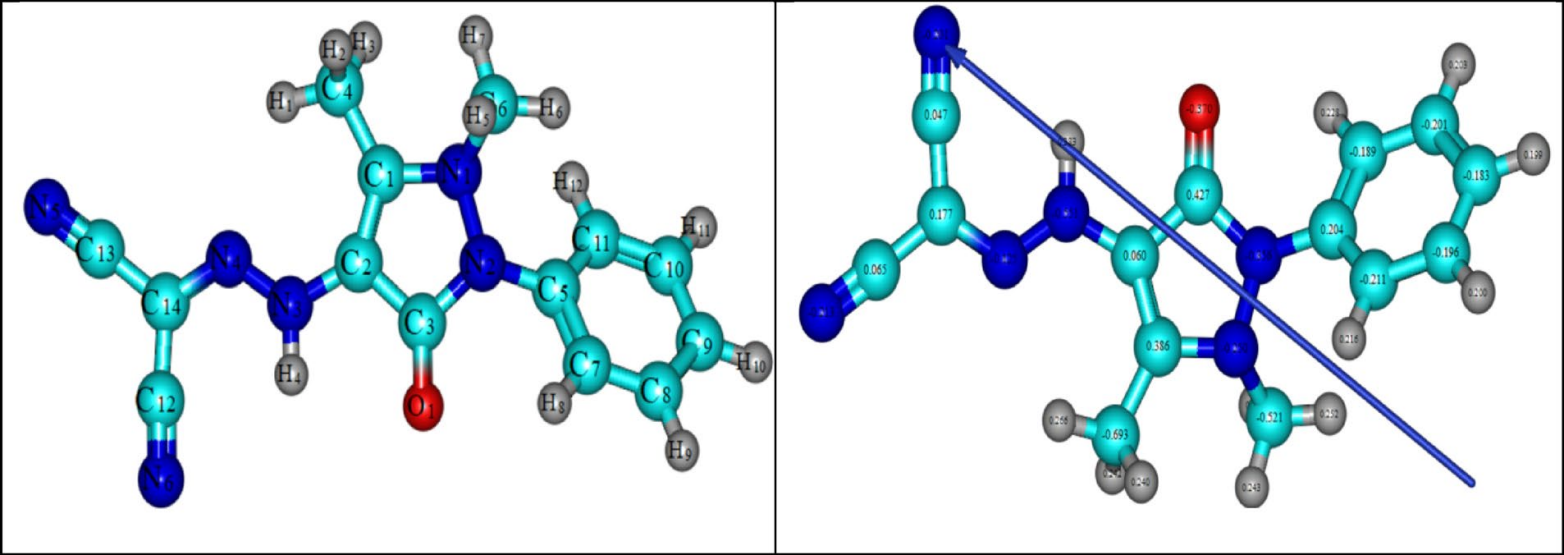
The optimized structure of ligand, the vector of the dipole moment, and the natural charges on active centers of ligand B by density function B3LYP/6–311 + g(d,p).

The ligand B optimized structures, which have the lowest energy configurations, are displayed in Fig. 10. Natural Bond Orbital Analysis (NBO) natural charges reveal the more negative active sites: O1 (− 0.370), N1 (− 0.250), N2 (− 0.356), N3 (− 0.551), N4 (− 0.125), N5 (− 0.201), and N6 (− 0.213). Metal ions create a six-membered bidentate chelate ring by coordinating with O1 and N4 atoms.

#### Molecular DFT calculation of metal complexes

**Fig. 11:**
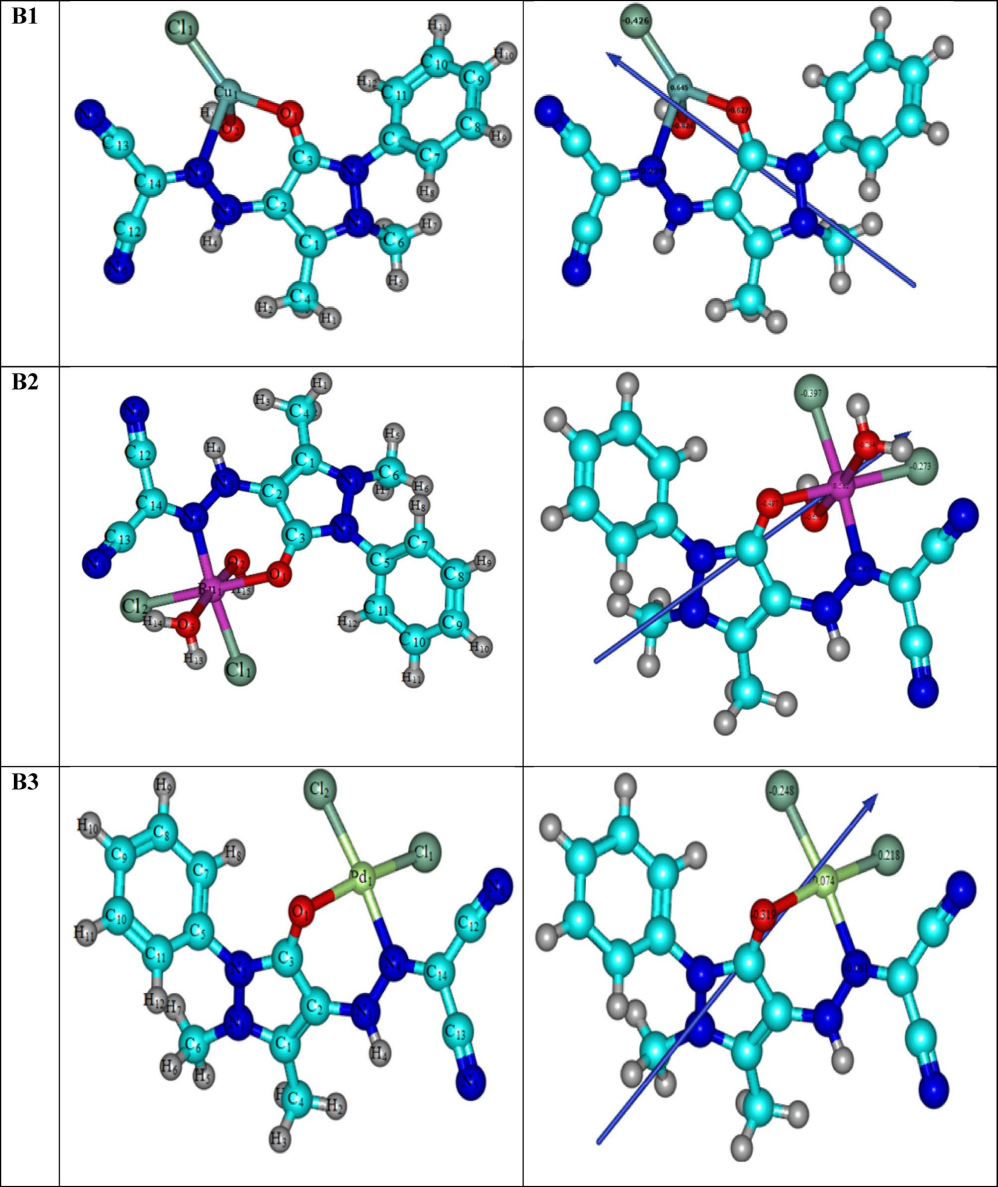
3D optimized structure of the [Cu(HL)Cl(OH)](**B1**), [Ru(HL)Cl_2_(OH)] (**B2**) and [Pd(HL)Cl_2_] (**B3**) complexes.

Using the B3LYP/6-311G + (dp) basis sets, the geometry of the Cu(II), Ru(III), and Pd(II) complexes was optimized for all atoms except metal ions^39–41^. When HL coordinates with the metal atom, some bond lengths become slightly longer or shorter than in the free ligand. As seen in Fig. 11, metal complexes were identified to have tetrahedral, square planar, and octahedral geometry for Cu(II), pd(II), and Ru(III) complexes, respectively. According to the NBO-analysis, the coordinated atoms’ natural charges are Cu (+ 0.645), O1 (− 0.627), O2 (− 0.824), Cl1 (− 0.426), and N4 (− 0.229), whereas The coordinated atoms in the Ru(III) complex have the following natural charges: Ru (+ 0.502), O1 (− 0.474), O2 (− 0.675), O3 (− 0.754), N4 (− 0.295), Cl1 (− 0.397), and Cl2 (− 0.476). The inherent charges on the coordinated atoms of the Pd(II) complex are Pd (+ 0.074), O1 (− 0.319), Cl2 (− 0.248), Cl1 (− 0.218), and N4 (− 0.081).

#### HOMO/LUMO energy evaluation

**Fig. 12:**
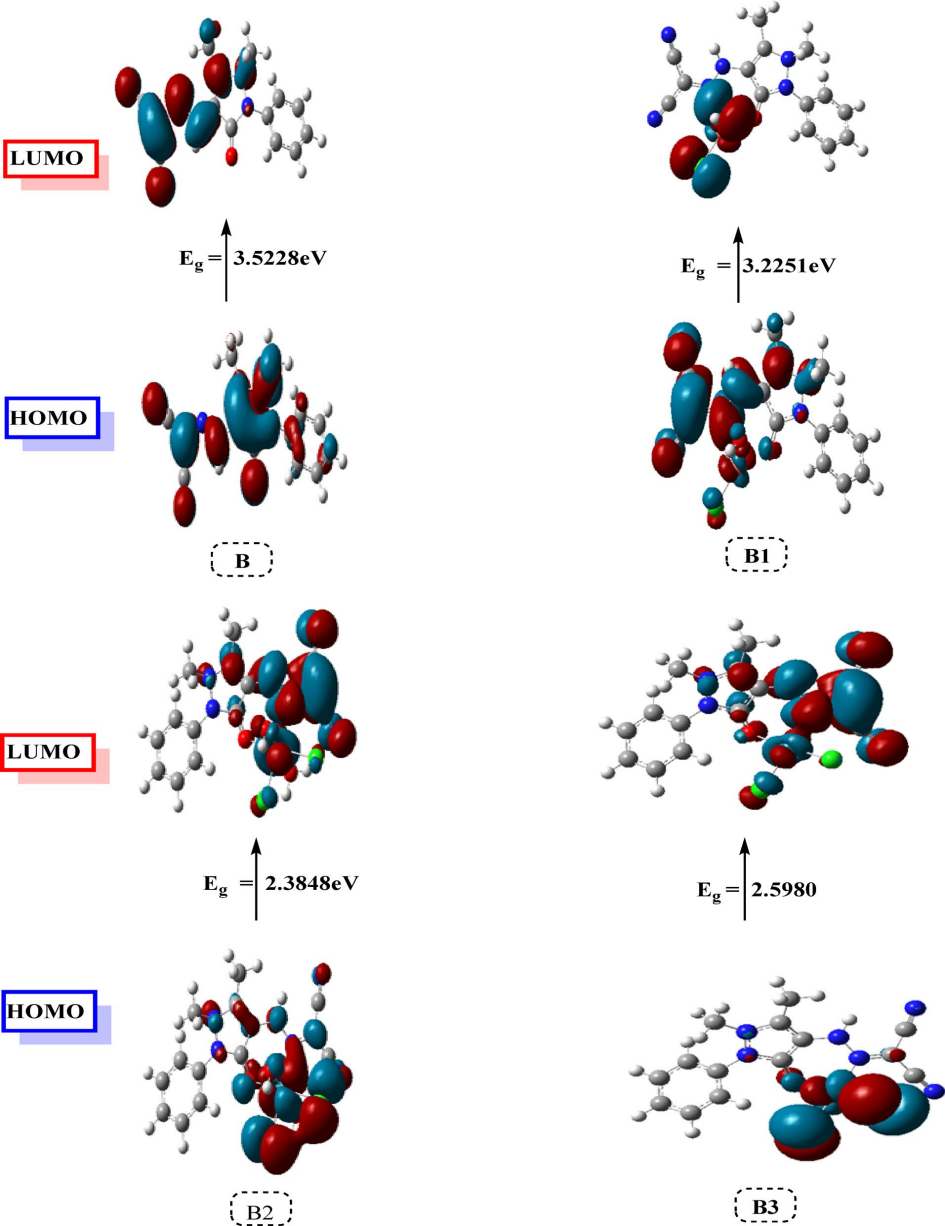
MO and their energies for ligand (**B**), Cu(II)complex(**B1**), [Ru((III) complex(**B2**) and Pd(II)complex(**B3**).

HOMO/LUMO energy evaluation is a crucial technique for examining the behavior of compounds (Fig. 12). The LUMO energy indicates the compound’s capacity to take on electrons, whereas the HOMO energy indicates its capacity to give them away^42,43^. Cu(II), Ru(III), and Pd(II) complexes have lower total energies than the free ligand, suggesting that they are more stable. Molecular orbitals are also used to estimate other chemical parameters are listed in Table 6^23^. A hard molecule with poor reactivity was said to be produced by a molecule with a high energy gap (Eg) value. A higher softness (S) value for the Pd(II) complex indicates a higher level of chemical reactivity. To support the theoretical results, the calculated DFT parameters were linked with the experimental cytotoxicity data. The relationship between energy gap, softness, dipole moment, and electrophilicity with the IC₅₀ values is highlighted in the cytotoxicity section.

**Table 6:** Calculated properties of ligand B, complexes (B1, B2 and B3).

Property	B	B1	B2	B3
The total energy E (a.u.)	− 944.565	− 3120.711	− 2110.975	− 1100.886
HOMO (eV)	− 6.298	− 6.026	− 5.900	− 6.299
LUMO (eV)	− 2.775	− 2.801	− 3.515	− 3.701
E_g_ = E_LUMO_–E_HOMO_ (eV)	3.522	3.225	2.384	2.598
Dipole moment (Debye)	9.319	16.274	15.976	19.500
Ionization potential I = −E_HOMO_	6.298	6.026	5.900	6.299
Electron affinity A = −E_LUMO_	2.775	2.801	3.515	3.701
Electronegativity χ = (I + A)/2	− 4.536	− 4.413	− 4.708	− 5.000
Chemical hardness η = (I−A)/2	1.761	1.612	1.192	1.299
Chemical softness S = 1/2η	0.567	0.620	0.838	0.769
Chemical potential μ = −χ	4.536	4.413	4.708	5.000
Electrophilicity ω = μ^2^/2η	5.842	6.040	9.295	9.622

#### Electrostatic potential map (MEP)

**Fig. 13:**
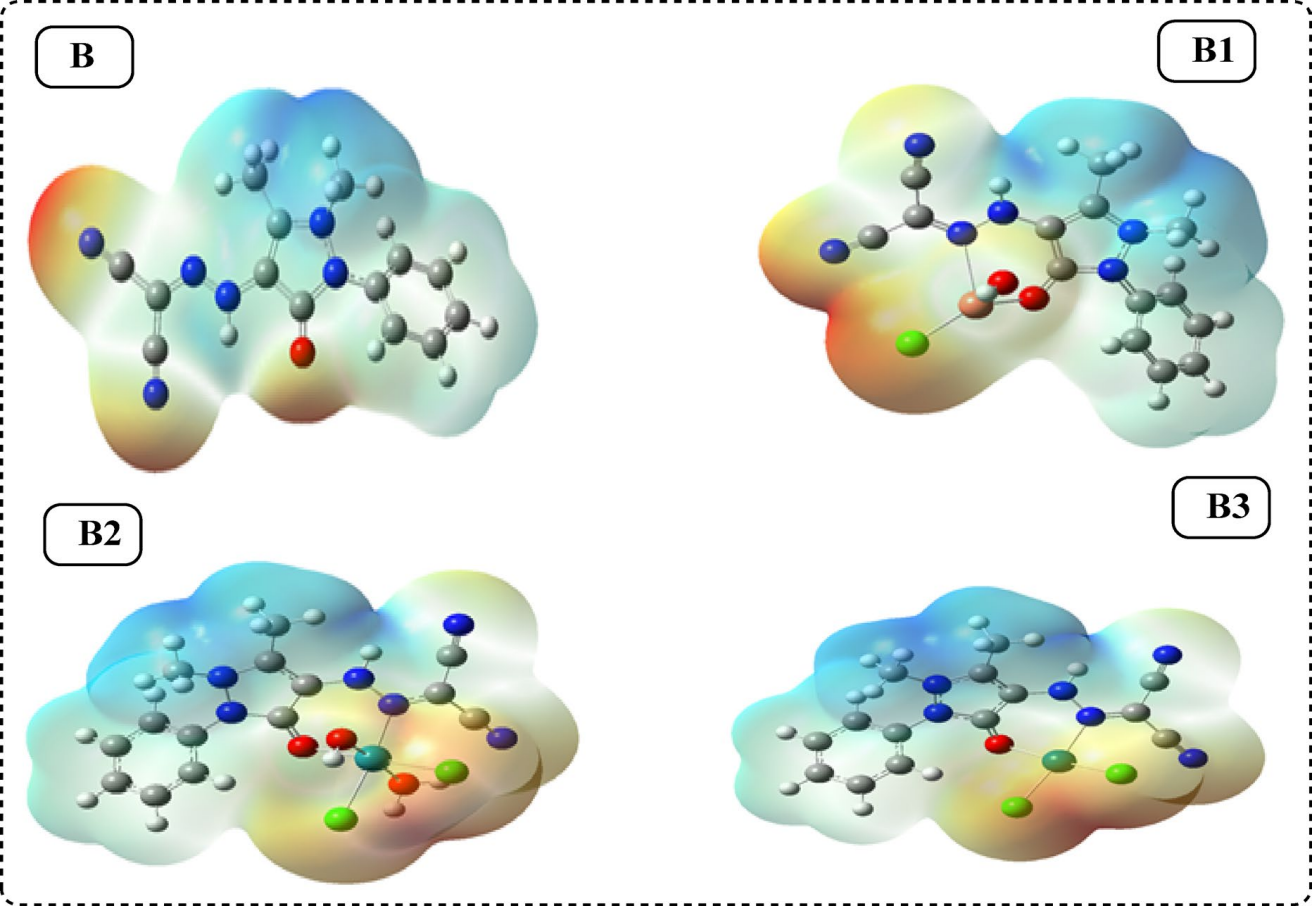
Molecular electrostatic potential (MEP) surface of ligand (**B**), Cu(II)complex(**B1**), [Ru((III) complex(**B2**) and Pd(II)complex(**B3**).

The distribution, size, structural structure, and dipole moments of the electrostatic potential are displayed by the total electron density surface^23^, which serves as the basis for the electrostatic potential mapping. The MEP for the ligand under investigation and its complexes is shown in Fig. 13. The MEP surface’s red, blue, yellow, and green colour zones stand for electron-rich, electron-poor, moderately electron-poor, and neutral zones, respectively. In the MEP, electronegative atoms (such as oxygen and nitrogen) have negative potential regions, whereas hydrogen atoms have positive potential zones. The MEP surfaces were dominated by the green area, which represents a potential that lies in the middle region of the red and dark blue colour spectrums.

### Antibacterial bioassay

**Fig. 14:**
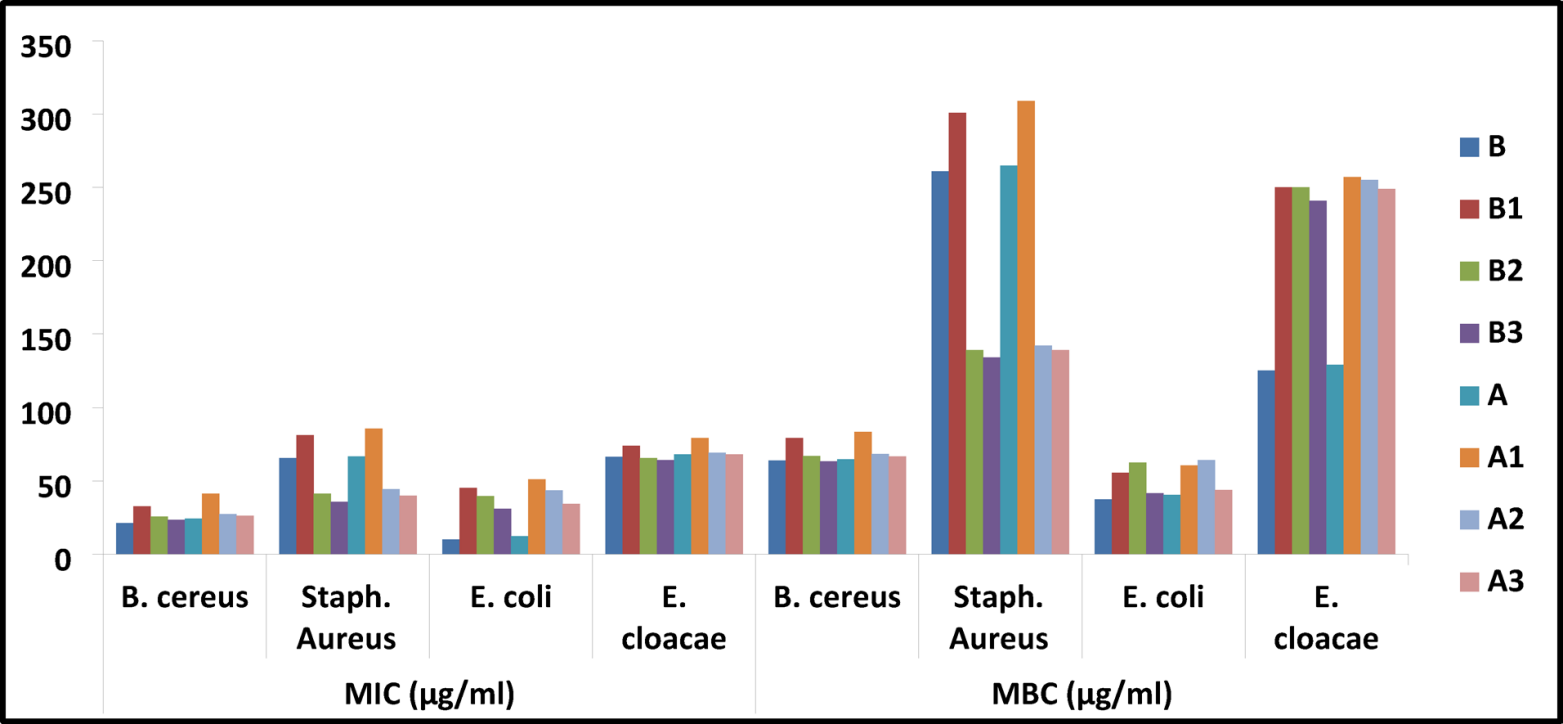
The antibacterial activity of all studied compounds at a concentration of 10 mg ml ^−1^ in relation to gentamycin and ampicillin as standard medications.

In this investigation, the antibacterial activity of HL and chelates in case of non-irradiated and irradiated states was assessed using the agar diffusion technique against Gram−ve bacteria *P. vulgaris* and *E. coli* as well as Gram + ve bacteria *S. aureus* and *B. subtili*^44^. The antimicrobial evaluation of the ligand (HL) and its Cu(II), Ru(III), and Pd(II) complexes before and after irradiation is presented in Table S1 and Fig. 14. The data revealed that the minimum inhibitory concentration (MIC) values generally increased after irradiation for all tested microorganisms, which clearly indicates a decrease in antimicrobial activity. For instance, the MIC of the free ligand against *B. cereus* increased from 21.23 µg/ml before irradiation (B) to 24.12 µg/ml after irradiation (A). A similar trend was observed for the Cu(II) complex, where MIC values against *E. coli* rose from 45.2 µg/ml (B1) to 51.11 µg/ml (A1), and for the Ru(III) complex, where the MIC against *Staphylococcus aureus* increased markedly from 125 µg/ml (B2) to 129 µg/ml (A2). Likewise, the Pd(II) complex showed an increase in MIC values after irradiation (e.g., from 30.9 to 34.18 µg/ml against *E. coli*). These findings are consistent with the corresponding minimum bactericidal concentration (MBC) values, which also increased after irradiation, confirming the reduction in bactericidal efficacy. Overall, the results demonstrate that irradiation negatively affected the antimicrobial potency of the ligand and its complexes. This decrease can be explained by structural or stability changes that occur after irradiation. Such changes may lower the ability of the complexes to interact with microbial cells, or lead to the formation of less active species. In short, irradiation had a negative effect on the antimicrobial performance of the ligand and its complexes. The antibacterial activity of the tested samples was further evaluated by calculating the MBC/MIC ratios, which are commonly used to distinguish between bactericidal (MBC/MIC ≤ 4) and bacteriostatic (MBC/MIC > 4) effects. Across all tested bacteria, the majority of the samples exhibited MBC/MIC values within the bactericidal range, particularly against *Bacillus cereus* and *Staphylococcus aureus*, where ratios were generally between 2.0 and 4.0. For instance, samples B, A, B1, and A1 displayed strong bactericidal potential against *B. cereus* (ratios 2.0–3.0) and *S. aureus* (ratios 3.6–4.0). Similarly, the activity against *E. coli* was predominantly bactericidal, with most ratios falling between 1.1 and 3.7, indicating effective killing rather than mere growth inhibition. In contrast, the activity against *Enterobacter cloacae* varied more widely, with samples B and A showing low ratios (~ 1.9, strongly bactericidal), while others such as B1, A1, B2, and A2 approached values around 3.2–4.0, still within the bactericidal threshold but less potent compared to other bacteria. Collectively, these results suggest that the tested compounds possess broad-spectrum bactericidal activity, with particularly strong effects against Gram-positive bacteria (*B. cereus* and *S. aureus*) and notable efficiency against *E. coli*. In general, metal complexes outperformed free ligands in terms of antimicrobial activity. This improvement is commonly explained by the chelation theory, which states that coordination between ligand and metal ions reduces the polarity of the metal centre and allows for partial sharing of positive charge with the donor groups^45^. However, in this study, the opposite was observed in the majority of results: the ligand showed higher activity than its complexes against the tested bacteria and fungi. This reduction in activity upon complexation can be explained by several factors. First, the larger size and higher molecular weight of the complexes compared to the ligands may hinder their diffusion through the bacterial cell wall, especially in Gram-negative bacteria with complex outer membranes. Second, chelation can sometimes reduce the availability of active functional groups in the ligand that are directly responsible for antimicrobial action, thereby diminishing their effectiveness. Moreover, the formation of stable complexes may limit the release of free metal ions or active moieties necessary for optimal interaction with microbial targets. These factors together suggest that while metal complexation generally enhances lipophilicity and improve cell penetration; in certain cases it can reduce biological activity if steric hindrance or reduced accessibility of active sites outweighs these benefits.

### Cytotoxicity

**Fig. 15:**
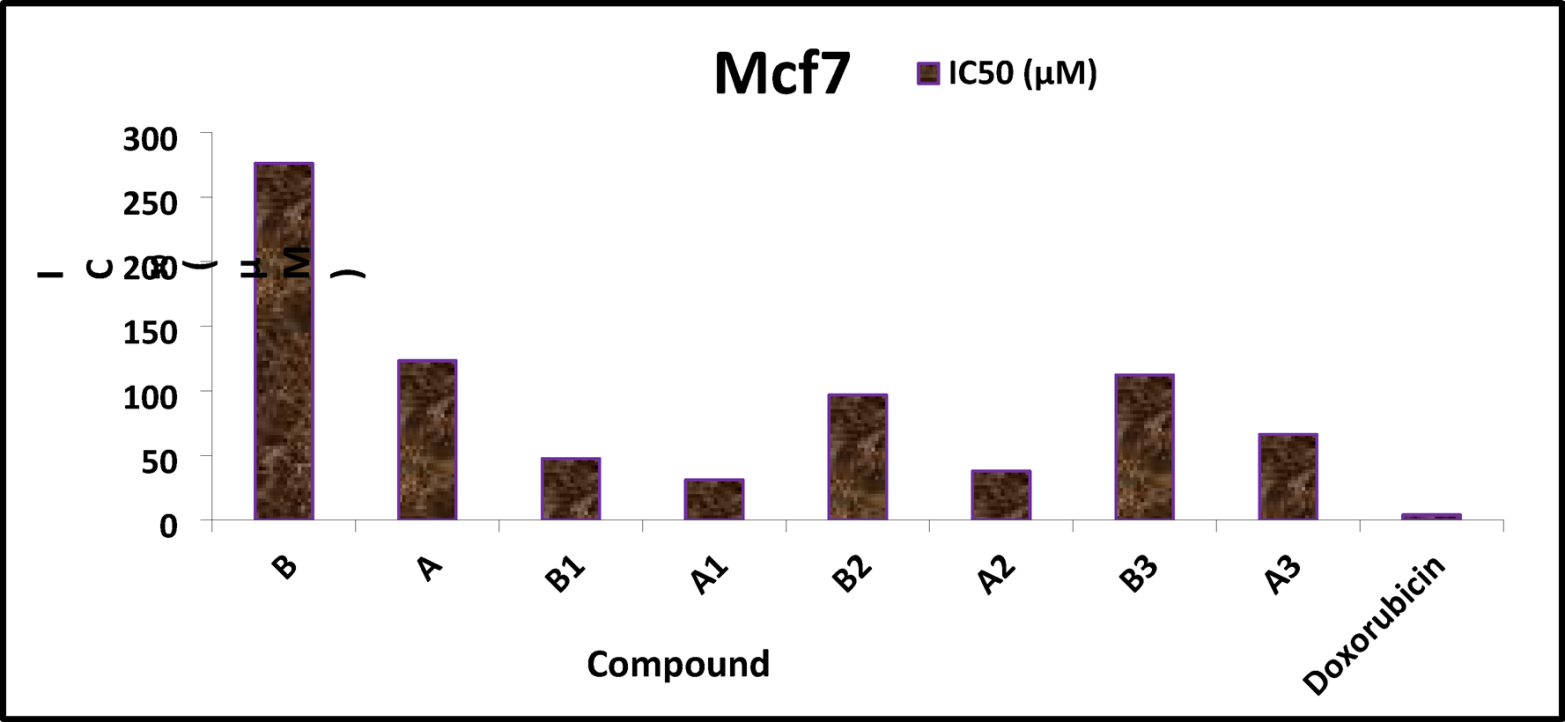
IC_50_ values of all investigated compounds against MCF7.

The **in vitro** study evaluated the cytotoxic effects of ligands (B, A) and their metal complexes—Cu(II) (B1, A1), Ru(III) (B2, A2), and Pd(II) (B3, A3)—on breast cancer cells before and after irradiation (Table S2, Fig. 15). All tested compounds exhibited measurable cytotoxicity, which generally increased after chelation. Among them, the Cu(II) complex after irradiation (A1) showed the lowest IC_50_ value (31.11 μg/ml), followed by the Pd(II) (A3) and Ru(III) (A2) complexes. The IC_50_ values for the free ligands and their complexes were: ligands (B, A) = 276.19 and 123.14 μg/ml; Cu(II) (B1, A1) = 47.57 and 31.11 μg/ml; Ru(III) (B2, A2) = 112.35 and 66.32 μg/ml; and Pd(II) (B3, A3) = 97.03 and 37.94 μg/ml. In comparison, doxorubicin, the reference drug, exhibited much stronger activity with an IC_50_ of 4.00 μM^12^. The sequence of activity was: doxorubicin < A1 < A2 < B1 < A3 < B2 < B3 < A < B. While the IC_50_ values of the complexes are considerably higher than doxorubicin and therefore indicate lower potency, their enhanced activity compared to the free ligands suggests that chelation improves cytotoxic properties. This improvement may result from their ability to interact with DNA and/or promote ROS generation, mechanisms commonly reported for metal-based anticancer agents^46^. The relatively higher activity of the Cu(II) complexes, particularly A1, may be linked to their capacity to modulate apoptotic pathways as previously described^47^. Thus, although these complexes cannot rival the clinical efficacy of doxorubicin, their post-irradiation enhancement indicates potential as lead structures for further optimization in anticancer drug development. Moreover, the cytotoxicity tests showed a different trend. After irradiation, both the ligand and its complexes became more active against tumor cells, as seen from the lower IC₅₀ values. This opposite effect compared with the microbial results suggests that irradiation alters the compounds in a way that favors their action on cancer cells. The comparison between the calculated DFT parameters and the cytotoxicity results gives a clear view of the structure–activity relationship of the studied compounds. The Cu(II) complex (A1), which showed the strongest activity after irradiation (IC₅₀ = 31.11 μg/ml), is characterized by a relatively small HOMO–LUMO gap (3.23 eV) and higher softness (0.62 eV^−1^), supporting its higher reactivity and stronger interaction with biological targets. The Pd(II) complex (A3) also displayed notable activity (IC₅₀ = 37.94 μg/ml), in line with its high dipole moment (19.5 D) and electrophilicity index (9.62 eV), both of which favor effective binding to biomolecules such as DNA and proteins. The Ru(III) complex (A2) showed a moderate effect (IC₅₀ = 66.32 μg/ml), which can be explained by its narrow gap (2.38 eV) and the highest softness value (0.84 eV^−1^). On the other hand, the free ligand, with the largest HOMO–LUMO gap (3.52 eV) and lowest softness (0.57 eV^−1^), was the least active (IC₅₀ = 123–276 μg/ml). Overall, these results indicate that reduced energy gaps, higher softness, and increased dipole moments are closely linked with stronger cytotoxic activity, providing a theoretical basis for the observed IC₅₀ values.

### Docking analysis

Docking studies were performed to explain the interaction of the prepared compounds with their biological targets^48^. The compounds (HL, B1, B2, and B3) were docked with penicillin-binding protein (PBP, 1NJ4)^49–52^, which plays a vital role in bacterial cell wall synthesis, and with the HER2 receptor (3PP0)^53^, a protein involved in cancer development.

**Fig. 16:**
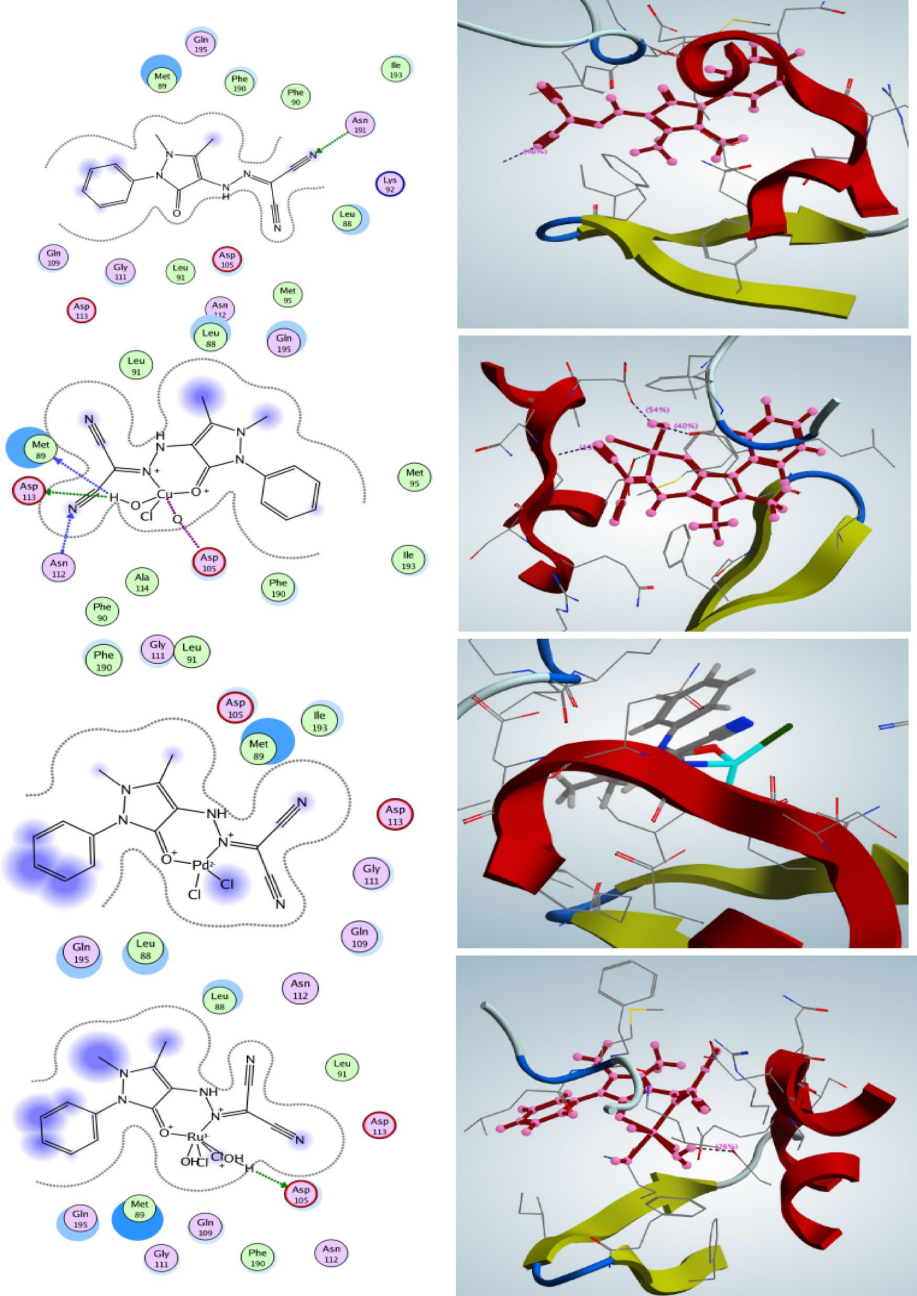
2D and 3D Diagrams of all synthesized compounds interaction with the penicillin-binding protein PBP enzyme interaction.

The free ligand (HL) showed only weak binding with both proteins. After complexation with metals, however, the binding became stronger and more stable. For 1NJ4, the best result was obtained with **B1** (−8.81 kcal/mol), which interacted with important residues such as Asn-191,Asp-113, Asn-112, Met-89, and Asp-105. Compounds B2 and B3 also showed good affinities (−8.22 and −8.20 kcal/mol), with Asp-105 acting as a common interaction site (Table 7 and Fig. 16). These findings are consistent with the enhanced antibacterial effect of the complexes. The docking results with PBP (1NJ4) agree well with the experimental findings, showing that the predicted binding strengths reflect the observed antibacterial activity.

**Table 7:** Binding affinities of the synthesized compounds with PBP (1NJ4) and HER2 (3PP0) along with RMSD values.

Enzymes	Compounds	Binding energy (kcal/mol, RMSD)	Involved amino acids	Type of interaction
PBP (1NJ4)	HL	− 4.47(1.20)	Asn-191	Sidechain donor
	B1	− 8.81(1.44)	Asp-113	Sidechain acceptor
			Met-89	Backbone acceptor
			Asn-112	Backbone donor
			Asp-105	Metal contact
	B2	− 8.22(1.01)	Asp-105	Sidechain acceptor
	B3	− 8.20(1.43)–6.39(1.64)	Asp-105	Silvent contact
			Met-89	
HER2 (3PP0)	HL	− 6.27(1.18)	–	Solvent contact
	B1	− 6.11(1.01)	–	Solvent contact
	B2	− 7.70(1.17)	Asp-105	Sidechain acceptor
	B3	− 6.17(1.31)	Ser-728	Silvent contact

**Fig. 17:**
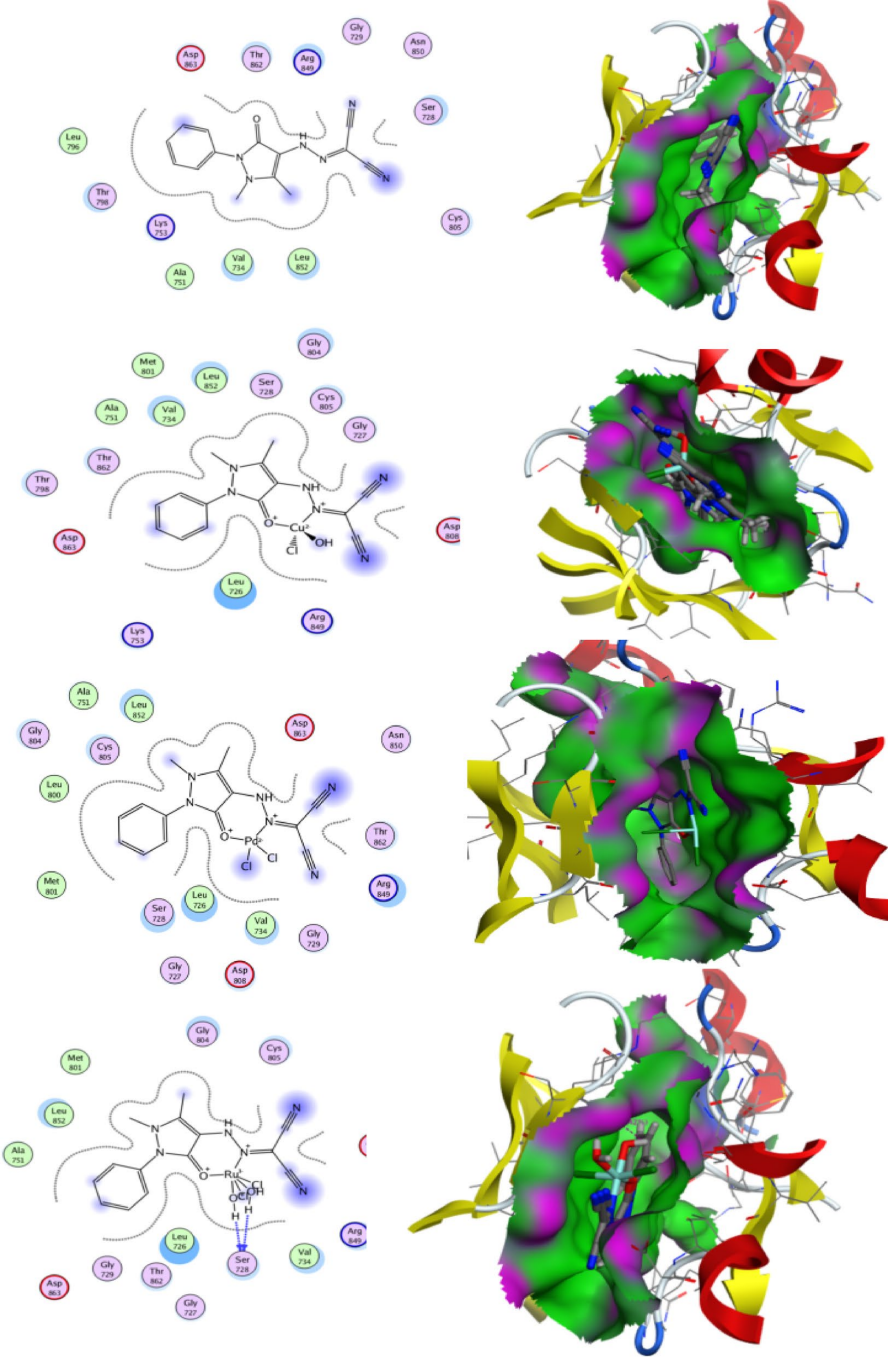
2D and 3D Diagrams of human epidermal growth factor 2 (HER2) interaction with the synthesized compounds.

Abnormal signaling from the ErbB family members, particularly human epidermal growth factor 2 (HER2) and epidermal growth factor receptor (EGFR), is associated with numerous human cancers, with HER2 expression serving as a predictor for disease recurrence and prognosis. Small molecule kinase inhibitors targeting EGFR as well as both HER2 and EGFR have been authorized for cancer treatment. Consequently, the interaction of the synthesized compounds with the studied HER2 provides insight into the suppression of the epidermal growth factor receptor. Table 7 and Fig. 17 indicate the scoring energy values with a negative sign to show the strong affinity of our compounds for the tested protein. Thus, these compounds are regarded as potent medications to prevent the emergence and development of cancer.

In the case of HER2, HL and B1 displayed moderate binding, while B2 gave the strongest interaction (−7.70 kcal/mol) through Asp-105. B3 interacted with Ser-728 but with lower affinity. Taken together, the results confirm that metal coordination improves the binding profile of the ligands. **B1** appears more effective against bacterial PBP, whereas **B2** shows selectivity toward HER2, pointing to a potential dual role as antibacterial.

## Conclusion

This work used a variety of spectroscopic and structural methods to completely describe and create novel Cu(II), Ru(III), Pd(II) complexes based on N-(1,5-dimethyl-3-oxo-2-phenyl-2,3-dihydro-1H-Pyrazol-4-yl)carbonohydrazonoyl dicyanide ligand. According to the results of thermal analysis, FTIR, molar conductivity and elemental analysis, the complex produced with a molar ratio of 1:1 M:L. DFT supported the proposed geometric structures. All compounds are prepared in the semi-crystalline form as indicated by the XRD and SEM techniques. All compounds showed inhibition for the bacterial growth and the breast cancer cell line specially Cu(II) complex. The anticancer activity was improved by gamma radiation process. The molecular docking supported the biological results.

## Supplementary Information

Below is the link to the electronic supplementary material.

Supplementary Material 1

## Data Availability

Data is available on request Contact: Safaa S. Hassan, hsafaa@sci.cu.edu.eg.
