# Supplementary material for: Effect of gamma irradiation on the Cu(II), Ru(III) and Pd(II) complexes of azo antipyrine moiety and their biological applications
Source: Sci Rep. 2025 Oct 28;15:37728. doi: 10.1038/s41598-025-22252-3 (PMC12569019; doi:10.1038/s41598-025-22252-3)
Supplement: Supplementary file 1 — Supplementary Material 1 [file 41598_2025_22252_MOESM1_ESM.docx]

**Effect of gamma irradiation on the Cu(II), Ru(III) and Pd(II) complexes of azo antipyrine moiety and their biological applications**

**Ehab M. Abdalla ^a^*, Samar A. Aly ^b^*, Ayman E. Hassan ^b^, Safaa S. Hassan ^c^*, Emad F. Newair ^d^ and** **Shimaa Hosny ^a^**

^a*^ Chemistry Department, Faculty of Science, New Valley University, Alkharga 72511, Egypt.

^b*^ Department of Environmental Biotechnology, Genetic Engineering and Biotechnology Research Institute, University of Sadat City 32958, Egypt.

^c^ Chemistry Department, Faculty of Science, Cairo University, Giza 12613, Egypt.

^d^ Chemistry Department, Faculty of Science, Sohag University, Egypt.

**Corresponding authors: Email: Ehababdalla99@sci.nvu.edu.eg Tel.: +201008275126*

*E-mail:* [*samar.mostafa@gebri.usc.edu.eg*](mailto:samar.mostafa@gebri.usc.edu.eg) *Tel.: +201063936729*

*E-mail: hsafaa@sci.cu.edu.eg*

**Section S1. Materials and Physical measurements**

All the substances used, containing 4-amino antipyrine, Malononitrile, Sodium acetate and CuCl_2_.2H_2_O, RuCl_3_.3H_2_O and K_2_PdCl_4_ were acquired from Fluka and Sigma-Aldrich Chemie (Germany). Commercially accessible pure ethanol and dimethylformamide were utilized without purification.

The characterization of ligand and Cu(II), Ru(III) and Pd(II)complexes was carried out utilizing the various spectroscopic techniques: Elemental analyses (C, H, Cl and N) were performed by Micro analytical unit of the Cairo University, Egypt. Metal content was estimated using EDTA following standard methods. Mass spectrum for ligand and complex was run on Shimadzu-QP 2010 plus Mass Spectrometer, Microanalytical Laboratory, Faculty of Science, Cairo University, Egypt. The Fourier transform infrared absorption spectra of solid ligand and metal complex was performed by Micro analytical unit at Cairo University, Egypt, in the wavelength range (4000-400 cm^-1^) using KBr discs using a Nenexeus-Nicolidite -640-MSA FT-IR infrared spectrophotometer. The electronic spectra (ultraviolet/visible) were recorded using a Perkin Elmer Lambada 330 spectrophotometer. The electronic spectra of Dimethyl Formamide (DMF) solution of both the ligand and metal complexes were recorded in 1cm quartz cells. The molar conductivity measurements were determined in N, N′ Dimethyl- formamide (DMF) solution at (10^-3^M) using a tacussel conductimeter type CD6N.

Thermal analysis (TG/DTG) was obtained out by using a Shimadzu DTA/TG-50 crucibles at central lab, Tanta university, Egypt. Thermal Analyzer with heating rate of 10 ^o^C/min in nitrogen atmosphere with a following rate 20 mL/min in the range of ambient temperature up to 800 ⁰C using platinum crucibles. The electron impact (EI) mass spectrum for the ligand was run on Shimadzu-QP 2010 plus Mass Spectrometer, Micro analytical Laboratory, Faculty of Science, Cairo University, Egypt. X-ray powder diffraction analyses of solid samples were measured using APD 2000 PROModel GNR-X-ray Diffractometer at (Central lab., Tanta University, Egypt). X-ray diffractograms gives computer control formally finished by PHILIPS®MPDX´PERT X-ray diffratometer ready with Cu radiation CuKα (λ=1.540 56 Å). The x´pertdiffractometer has the Bragg- Brentano geometry. The x-ray tube used was a copper tube operating at 40 KV and 30 mA. The scanning range (2θ) was 5–90° with step size of 0.050° and counting time of 2 s/step. Quartz was used as the standard material to accurate for the instrumental expansion. This identification of the complexes was done by a known method. From the fit identified Scherrer formula, the average crystallite size (D) is

D = (Kλ /β cos θ)

Where:

λ is the X- ray wavelength in the nanometer, K is factor related to crystallite shape, with a value about 0.9 and ß is the peak width at half maximum height. The value of ß in the 2θ axis of diffraction shape must be in radians. The θ is the Bragg angle and be able to in radians since the Cos θ compatible with the same number.

Surface morphological study was recorded by taking scanning electron microscopy (SEM) images for unirradiated (B, B1 ,B2, B3) and irradiated (R, R1, R2, R3) samples of compounds ligand, [Cu(HL)Cl(OH)].H_2_O, [Ru(HL)Cl_2_(OH)(H_2_O)].H_2_O and [Pd(HL)Cl_2_].2H_2_O in JEOL model JSM-5500 equipment.

Energetic γ-irradiation exposure was undertaken using a γ-^60^Co unit at atomic energy establishment (AEE) at El-Nasr City , Egypt at an accumalated dose of 60 KGY where (1Mega rad=10KGY) in air.

**Section S2:Antibacterial activity**

The antimicrobial activity of the synthesized compounds was examined using the broth microdilution assay. Briefly, bacterial and fungal suspensions were prepared (≈10⁸ CFU/mL for bacteria and ≈10⁵ CFU/mL for fungi) in fresh medium. Serial dilutions of the compounds (starting at 0.5 mg/mL) were added to sterile microtiter plates and incubated for 24 h at 37 °C. MIC values were recorded as the lowest concentration showing no visible growth, while MBC values were determined by subculturing onto fresh agar plates. Penicillin G was used as standard antibacterial (Gram +) drug while Ciprofloxacin was used as standard antibacterial (Gram -) drug and Ketoconazole as Antifungal drug.

**Table S1.** The in-vitro antibacterial activity of ligand and its metal complexes before and after irradiation

| No | **MIC (µg/ml)** | | | | **MBC (µg/ml)** | | | |
| --- | --- | --- | --- | --- | --- | --- | --- | --- |
|  | **B. cereus** | **Staph. Aureus** | **E. coli** | **E. cloacae** | **B. cereus** | **Staph. Aureus** | **E. coli** | **E. cloacae** |
| B | 21.23 | 65.6 | 10.11 | 66.5 | 63.9 | 261 | 37.2 | 125 |
| A | 24.12 | 66.52 | 12.13 | 68.01 | 64.81 | 265 | 40.5 | 129 |
| B1 | 32.56 | 81.2 | 45.2 | 73.8 | 79.3 | 301 | 55.6 | 250 |
| A1 | 41.32 | 85.63 | 51.11 | 79.19 | 83.45 | 309 | 60.46 | 257 |
| B2 | 31.3 | 125 | 15.6 | 125 | 125 | 500 | 62.5 | 500 |
| A2 | 32.6 | 129 | 19.5 | 127 | 131 | 504 | 65.2 | 515 |
| B3 | 23.4 | 35.6 | 30.9 | 64.1 | 63.4 | 134 | 41.6 | 241 |
| A3 | 26.25 | 39.71 | 34.18 | 68.12 | 66.65 | 139 | 43.71 | 249 |

**Table S2:** Results of cytotoxic activity (IC_50_) of the ligand, Cu(II), Ru(III), and Pd(II) before and after irradiation against human MCF7cell line at different concentration levels.

| **Compound** | **IC_50_ (µM)** |
| --- | --- |
| **B** | 276.19 |
| **A** | 123.14 |
| **B1** | 47.57 |
| **A1** | 31.11 |
| **B2** | 112.35 |
| **A2** | 66.32 |
| **B3** | 97.03 |
| **A3** | 37.94 |
| **Doxorubicin** | 4.00 |

**Figure S1**: Mass fragmentation of ligand and metal complexes
